# CircPUM1 promotes hepatocellular carcinoma progression through the miR‐1208/MAP3K2 axis

**DOI:** 10.1111/jcmm.15998

**Published:** 2020-12-15

**Authors:** Yaqiong Zhang, Dongguo Wang, Tao Zhu, Jin Yu, Xiaoyu Wu, Weidong Lin, Minqi Zhu, Yingjie Dai, Jie Zhu

**Affiliations:** ^1^ Department of Clinical Laboratory Taizhou Central Hospital (Taizhou University Hospital) Zhejiang China; ^2^ Department of Clinical Lab Medicine Taizhou Municipal Hospital Affiliated with Taizhou University Zhejiang China; ^3^ Department of Plastic surgery Taizhou Central Hospital (Taizhou University Hospital) Zhejiang China; ^4^ The Health Management Centre Taizhou Central Hospital (Taizhou University Hospital) Taizhou China

**Keywords:** circPUM1, hepatocellular carcinoma, MAP3K2, miR‐1208

## Abstract

Hepatocellular carcinoma (HCC) is a common disease with a significant mortality, and there is no effective treatment for advanced patients. Growing evidence indicates that circRNAs are closely related to HCC progression, may be used as biomarkers and targets for the diagnosis and treatment of HCC. Recent researches have shown that circPUM1 may play an oncogene role in a variety of human cancers, but its role in HCC development has not been reported. Our study found that circPUM1 could promote the proliferation, migration and invasion of HCC cells in vitro. In addition, in vivo studies showed that circPUM1 could increase the development of HCC tumours and regulate the expression of EMT‐related proteins. Furthermore, we demonstrated that circPUM1 could promote the development of HCC by up‐regulating the expression of MAP3K2 via sponging miR‐1208. Our study suggested that circPUM1 may be a potential therapeutic target for HCC.

## 1 INTRODUCTION

Liver cancer is one of the most common malignant tumours in the world, which is the leading cause of cancer‐related death.^1^ Hepatocellular carcinoma (HCC) is a major subtype of liver cancer. In the early stage of HCC, surgical resection can be used and better therapeutic effect can be achieved. However, some patients were already in the advanced stage when they were found to have HCC. After treatment, there would be recurrence, metastasis and drug resistance, and the prognosis was extremely poor.^2^, ^3^ Therefore, it is necessary and urgent to study new diagnostic markers and therapeutic targets for HCC.

Circular RNAs (circRNAs) are a special novel type of endogenous non‐coding RNAs (ncRNAs), which form a covalently closed continuous loop and are highly expressed in the eukaryotic transcriptome. Recent research has shown that circRNAs can function as microRNA (miRNA) sponges and participate in post‐transcriptional regulation as an endogenous competitive RNA (ceRNA).^4^, ^5^ Increasing evidence indicates that the change of circRNA expression has a broad influence on biological characteristics of HCC. CircRNAs can act as oncogenes or tumour suppressors in HCC.^6^, ^7^, ^8^ For example, circFBXO11 could promote HCC development and oxaliplatin resistance via miR‐605/FOXO3/ABCB1 axis.^8^ CircABCB10 could suppress HCC progression via up‐regulating NRP1/ABL2 by acting as miR‐340‐5p/miR‐452‐5p sponge.^9^ CircMALAT1 could promote self‐renewal of HCC stem cells via inhibiting mRNA translation and sponging microRNA.^10^ Therefore, circRNAs may be potential therapeutic targets for HCC, and it is necessary to further study the role of circRNAs in the development of HCC.

Circular PUM1 RNA (circPUM1, has_circ_0000043) is derived from exonic back‐splicing of PUM1 gene. Previous studies showed that circPUM1 is highly expressed in lung adenocarcinoma cell lines and tissues.^11^ In addition, circPUM1 promotes proliferation, migration, invasion and apoptosis by inhibiting miR‐326, indicating that circPUM1 may be a target for the treatment of lung adenocarcinoma.^11^ Guan et al^12^ found that circPUM1 was up‐regulated in ovarian cancer tissues compared with normal ovaries. They proved that circPUM1 could promote the development and progression of ovarian cancer by sponging miR‐615‐5p and miR‐6753‐5p.^12^ Tumour‐derived exosomes are the mediators of tumour metastasis by creating a suitable environment for tumour survival, angiogenesis and invasion.^13^ Recent studies have shown that circRNAs can be transferred into exosomes. Guan et al^12^ demonstrated that circPUM1 was involved in tumour metastasis in the form of oncogenic exosomes. Zong et al^14^ reported that the expression level of CircPUM1 in endometrial carcinoma tissues was significantly higher than that in normal tissues. What's more, they found circPUM1 could promote endometrial cancer development via acting as the sponge of miR‐136 to regulate Notch3. In summary, circPUM1 may function as an oncogene in human cancers. However, the function of circPUM1 in HCC proliferation is unknown, and its role in the invasion, migration and metastasis of HCC has not been reported. Here, we studied the role of circPUM1 in HCC and its related mechanisms. Our research may provide theoretical support for the treatment of HCC.

## 2 MATERIALS AND METHODS

### 2.1 Cell culture

The Shanghai Tong Pai (Shanghai, China) and Cobioer (Nanjing, China) made the HCC cell lines available that included HCCLM3, MHCC97H, MHCC97L, SK‐HEP‐1, HUH7 and HEPG2. The Cobioer (Nanjing, China) provided the THLE‐3 normal human liver cell line. Maintaining a humidified atmosphere at 37°C and using the RPMI‐1640/DMEM, the cell lines were cultured with 5% CO_2_ while being supplemented with 10% FBS (Gibco, USA).

### 2.2 Cell transfection

The shRNA for the circRNA PUM1‐1 was developed by us. Subsequently, the transfection of the HCC cells was performed with the circPUM1 shRNA. Shangeng Biotech (Guangzhou, China) designed the CircPUM1 shRNA#1, shRNA#2 and shRNA #3 sequences. To construct circPUM1 knockdown plasmids, fragments targeting the circPUM1 junction sites were cloned into pLKO.1‐puro vector. Shangeng Biotech (Guangzhou, China) developed the miR‐1208 overexpression vector (miR‐mimic) and the negative control (miR‐NC). The HCC cells were transfected with miR‐NC or miR‐1208 mimic by applying Lipofectamine 3000 (Invitrogen). For the construction of the MAP3K2 siRNA, the HCC cells with miR‐1208 inhibitor were treated for 48 hours subsequently, to suppress the miR‐1208. Shangeng Biotech (Guangzhou, China) designed the pLCDH‐circPUM1 plasmid (oe‐circPUM1) for the overexpression of the genes. The negative control (NC or vector) was represented by the empty pLCDH vector.

### 2.3 qRT‐PCR

Total RNA was isolated from striatum by using RNAiso Plus (TaKaRa, Dalian, China) and was reverse‐transcribed to cDNA using a PrimeScript RT reagent kit (TaKaRa). Quantitative real‐time PCR was performed to quantify circRNA and mRNA levels using SYBR Premix Ex Taq (TaKaRa). The mRNA and circRNA expression was normalized by GAPDH. The miRNA‐X PCR assay kit (Clontech, USA) was used to quantify the miRNA expression. All experiments were performed according to the manufacturer's instructions. The expression of miRNA was normalized by U6. All of primers are shown in Table S1.

### 2.4 CCK‐8 assay

Being cultured for 0 ~ 72 hours, the seeding of the 5 × 10^3^ cells was carried out in 96‐well plates. Subsequently, the incubation was continued for 2 more hours after adding 10 μl of the CCK‐8 assay solution (Beyotime, China). A microplate reader (Bio‐Rad, USA) was used to assess the absorbance in each well.

### 2.5 Scratch assay

Till reaching around 80% confluence as a monolayer, the cells were seeded in 6‐well tissue culture plates. The monolayer cells were gradually and gently scratched with a fresh 1‐ml pipette tip (0 hours). To remove the residual serum and the detached cells after scratching, the wells were carefully washed thrice with PBS (Hyclone, USA). All the wells were refilled with a serum‐free fresh medium subsequently. After 48 hours, using a microscope (Leica, Germany), the cell migration was photographed. Then, the gap area was calculated on the computer. The methods refer to previous article.^12^

### 2.6 Transwell migration and invasion assays

Overall, the seeding of the 5 × 10^4^ cells along with 1% FBS medium was carried out by placing in a 24‐well plate, using an 8‐μm Matrigel‐coated (CORNING, 356231) membrane Transwell chamber (CORNING, 3422), or a pore membrane. To the lower chamber of the 24‐well plate, plasmid (2.5 μl/ml) or 10% FBS medium with siRNA (100 nM) was added after the attachment of the cell. As described earlier, the invaded or migrated cells were stained after 36 hours.^15^ Three microscopic fields were determined after photographing the stained cells. The cells stained in each graph were calculated by count tool function of Photoshop software.

### 2.7 RNA pull‐down assay

The RNA pull‐down assays were performed with the Pierce™ Magnetic RNA‐Protein Pull‐Down Kit according to the previously reported protocol.^16^ The biotinylated miR‐1208 and control probes were designed by RiboBio (Guangzhou, China).

### 2.8 Luciferase reporter assay

Ligation of the segments of MAP3K2 containing miR‐1208 and circRNA PUM1 binding sites was carried out respectively, into the pmirGLO vectors. With the mutating binding sites having miR‐1208, the construction of the mutant plasmids was performed. As per the guidelines of the manufacture, the HCCLM3 and MHCC97H cells were co‐transfected with the indicated reporter vectors and the NC or the miR‐1208 mimics employing Lipofectamine 3000 (Invitrogen, USA). Thereafter, following the manufacturer's guidelines, the luciferase activity was analysed applying the dual‐luciferase reporting kit (Promega, USA) after 48 hours of co‐transfection.

### 2.9 RNA immunoprecipitation (RIP) assay

Following the instructions, the RIP assays were conducted with the Magna RIP RNA‐Binding Protein Immunoprecipitation Kit (Millipore, USA). For the RIP assay (Cell Signaling Technology, USA), the Ago2 antibody was used. The q‐PCR detected the co‐precipitated RNA.

### 2.10 Western blot analysis

Applying the phosphatase inhibitor cocktail (1:100) with 2 mm PMSF and the RIPA buffer (Millipore, USA) containing protease the tissue or cell was lysed. The BCA protein assay kit (Pierce, USA) was used to determine the protein concentration. For this assay a total of 20 μg of protein was used. The protein was transferred to 0.45‐μm nitrocellulose membrane (Millipore, USA) after being separated with 10% SDS‐polyacrylamide gel electrophoresis (SDS‐PAGE).

The membrane was hybridized with a secondary antibody (1:4000, Abcam, USA) for 1 hour at room temperature subsequent to the overnight incubation at 4°C with the primary antibodies. The ECL system detected the protein signals. Listing of the primary antibodies was done as under: GAPDH (1:2000, Abcam, USA), P‐ERK (1:1000, CST, USA), ERK (1:1000, CST, USA), P‐P38 (1:1000, CST, USA), P38 (1:1000, CST, USA), P‐JNK (1:1000, CST, USA), JNK (1:1000, CST, USA), Vimentin (1:1000, CST, USA), N‐cadherin (1:300, Santa Cruz, USA), E‐cadherin (1:300, Santa Cruz, USA) and MAP3K2 (1:1000, Thermo Fisher, USA).

### 2.11 Xenograft experiments

The Shanghai SLAC Laboratory Animal Co. Ltd (Shanghai, China) supplied the BALB/c nude mice. Maintaining a specific pathogen‐free atmosphere, the mice were housed in individually ventilated cages at 23°C at daylight/dark cycles of 12 hours. For experiment purposes, the 5‐week‐old male BALB/c nude mice were segregated randomly into 2 separate groups. The sh‐circFOXP1 lentivirus or the negative control (sh‐NC) was injected subcutaneously into the nude mice (1 × 10^6^, 100 μl) for transfecting the MHCC97H cells. Every 7 days, the volume of tumours was measured (0.5 × length × width^2^). The Medical Experimental Animal Care Commission provided the pertinent approval for the performance of all the animal experiments.

### 2.12 Statistical analysis

The GraphPad PRISM 6 (GraphPad Software, Inc, La Jolla, CA) was used for the performance of all the statistical analyses. The mean ± standard deviation method was applied to determine the data. Student's *t* tests were conducted for the analysis of the comparisons between the two groups. Tukey's post hoc tests were applied, for analysing through one‐way ANOVAs, the comparative data between the multiple groups. The significant statistical value was considered to be *P* < .05.

## 3 RESULTS

### 3.1 circPUM1 promotes the proliferation HCC cells in vitro

**FIGURE 1:**
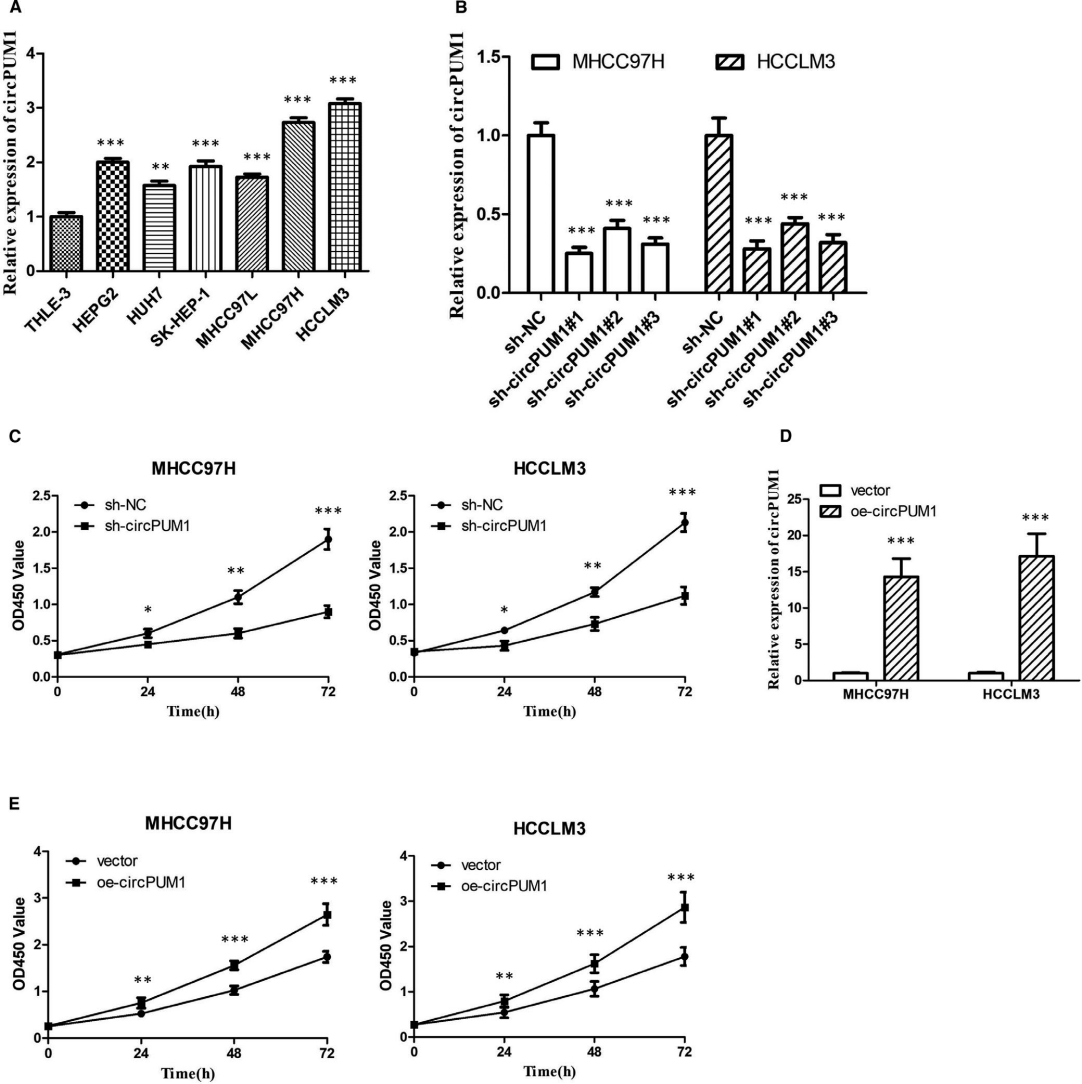
CircPUM1 promotes HCC cells proliferation. A, The expression of circPUM1 in different HCC cell lines was detected by qRT‐PCR assay. B, Knockdown effects of circPUM1 shRNA in MHCC97H and HCCLM3 cells. ***P < .001 vs THLE‐3 cells. C, The proliferation of MHCC97H and HCCLM3 cells with silencing circPUM1 was detected by CCK‐8 assay. D, qRT‐PCR assay showed the expression of circPUM1 in MHCC97H and HCCLM3 cells with overexpressing circPUM1. E, The proliferation of MHCC97H and HCCLM3 cells with overexpressing circPUM1 was detected by CCK‐8 assay. *P < .05, **P < .01, ***P < .001 vs sh‐NC or vector

To investigate the expression of circPUM1 in HCC, we performed qRT‐PCR in HCC cell lines and normal human liver cell line (THLE‐3). The result showed that the expression circPUM1 was the highest in MHCC97H, followed by HCCLM3 compared with THLE‐3 and other HCC cell lines (Figure 1A). We choose MHCC97H and HCCLM3 for the following experiment. To explore the role of circPUM1 in HCC, we used three circPUM1 shRNA to target and repress this circRNA. These siRNAs significantly decreased circPUM1 expression without decreasing the linear PUM1 mRNA level (Figure 1B). In addition, CCK‐8 assay showed that circPUM1 shRNA could inhibit HCC cell proliferation (Figure 1C). Sh‐circPUM1#1 showed the strongest inhibitory effect and was used in subsequent experiments. To further study the function of circRNA, we transferred circPUM1‐overexpressing plasmid (OE‐circPUM1) in MHCC97H and HCCLM3 cells (Figure 1D). Consistent with our previous studies, OE‐circPUM1 could promote HCC cells proliferation (Figure 1E).

### 3.2 circPUM1 promotes the migration and invasion HCC cells in vitro

**FIGURE 2:**
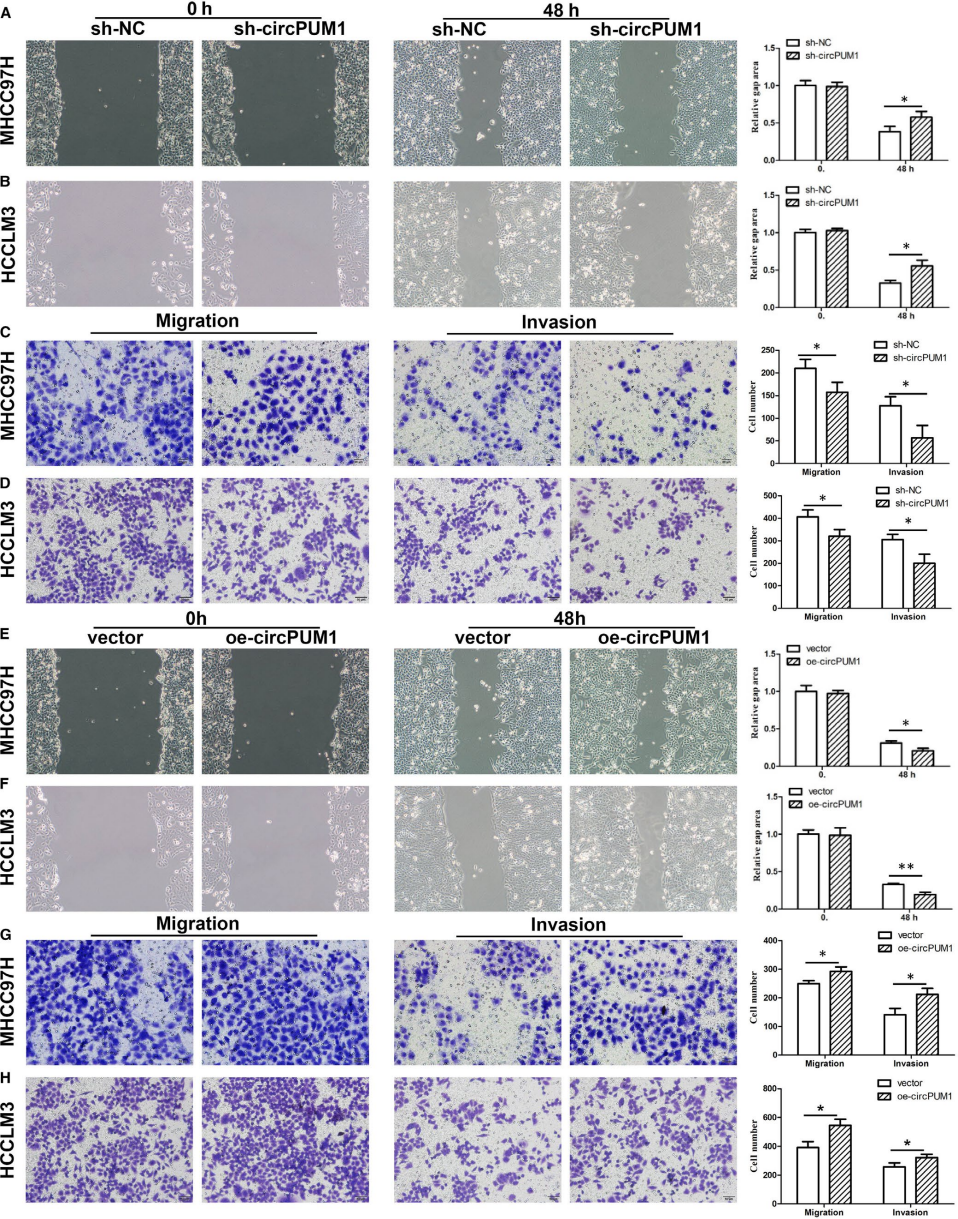
CircPUM1 promotes HCC cells migration and invasion. A and C, The migration of MHCC97H and HCCLM3 cells with repressing or overexpressing circPUM1 was detected by starch assay. B and D, Transwell assay was performed to detect the migration and invasion of MHCC97H and HCCLM3 cells with repressing or overexpressing circPUM1. *P < .05 vs sh‐NC or vector

Migration, invasion and metastasis are closely related to poor prognosis of HCC. Scratch assays were performed to detect the migratory capacity of HCC. Our results showed that si‐circPUM1 could inhibit MHCC97H and HCCLM3 cell migration (Figure 2A,B). In addition, Transwell assays further demonstrated that sh‐circPUM1 represses the migratory and invasive capacity (Figure 2C,D). Furthermore, overexpression of circPUM1 significantly enhanced the ability to promote HCC cells migration and invasion, corresponding to our previous results (Figure 2E,F). What's more, we found that circPUM1 could regulate the apoptosis of HCC cells (Figure S1A,B). Tumour metastasis involves multiple steps, among which invasion is the premise of metastasis, and epithelial‐mesenchymal transition (EMT) plays an important role in the process of tumour invasion and metastasis.^17^ Western blot results showed that sh‐circPUM1 significantly inhibited the expression of EMT‐related protein, such as N‐cadherin and vimentin, and enhanced the expression of E‐cadherin, indicating the progress of EMT was repressed (Figure S1C). Overexpression of circPUM1 further confirmed this result (Figure S1D).

### 3.3 circPUM1 acts as a molecular sponge to regulate miR‐1208 in HCC cells

**FIGURE 3:**
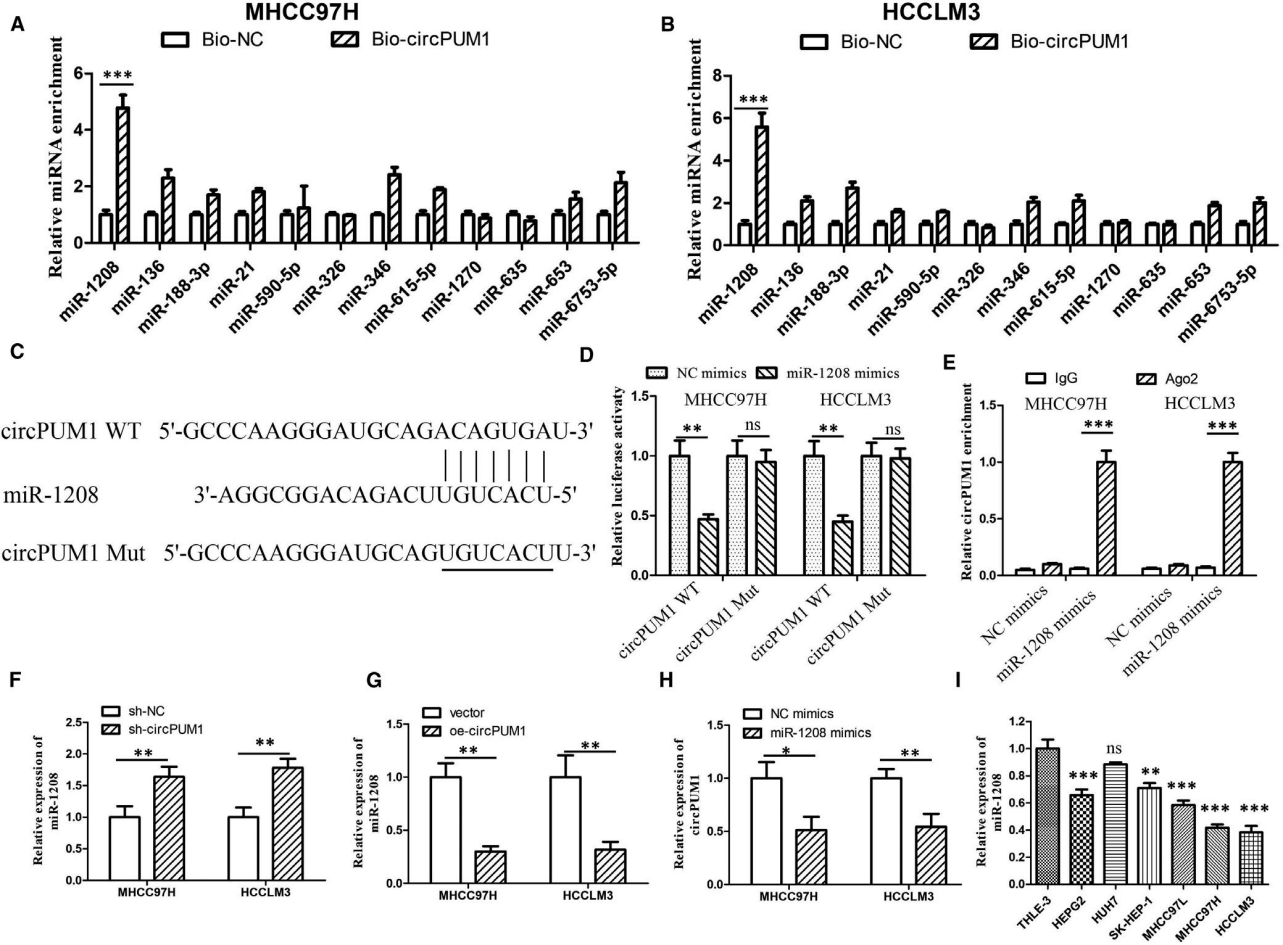
circPUM1 functions as a miR‐1208 sponge in HCC cells. A‐B, QRT‐PCR was performed to detect the Relative expression of circPUM1 putative binding miRNAs by Bio‐circPUM1 in HCC cells. C, Luciferase reporter assay analysis of the binding between miR‐1208 and predicted binding sites in circPUM1. B, Relative luciferase activity was determined by in MHCC97H and HCCLM3 cells. D, Anti‐AGO2 RIP was performed in MHCC97H and HCCLM3 cells after miR‐1208 mimics transfection. E‐F, Expression of miR‐1208 in MHCC97H and HCCLM3 cells after repressing or overexpressing circPUM1 was detected by qRT‐PCR. G. Expression of circPUM1 in MHCC97H and HCCLM3 cells with overexpressing miR‐1208 was detected by qRT‐PCR. I. The expression of miR‐1208 in different HCC cell lines was detected by qRT‐PCR assay. *P < .05, **P < .01, ***P < .01 vs Bio‐NC, sh‐NC, vector or NC mimics. Ns, no significant

**FIGURE 4:**
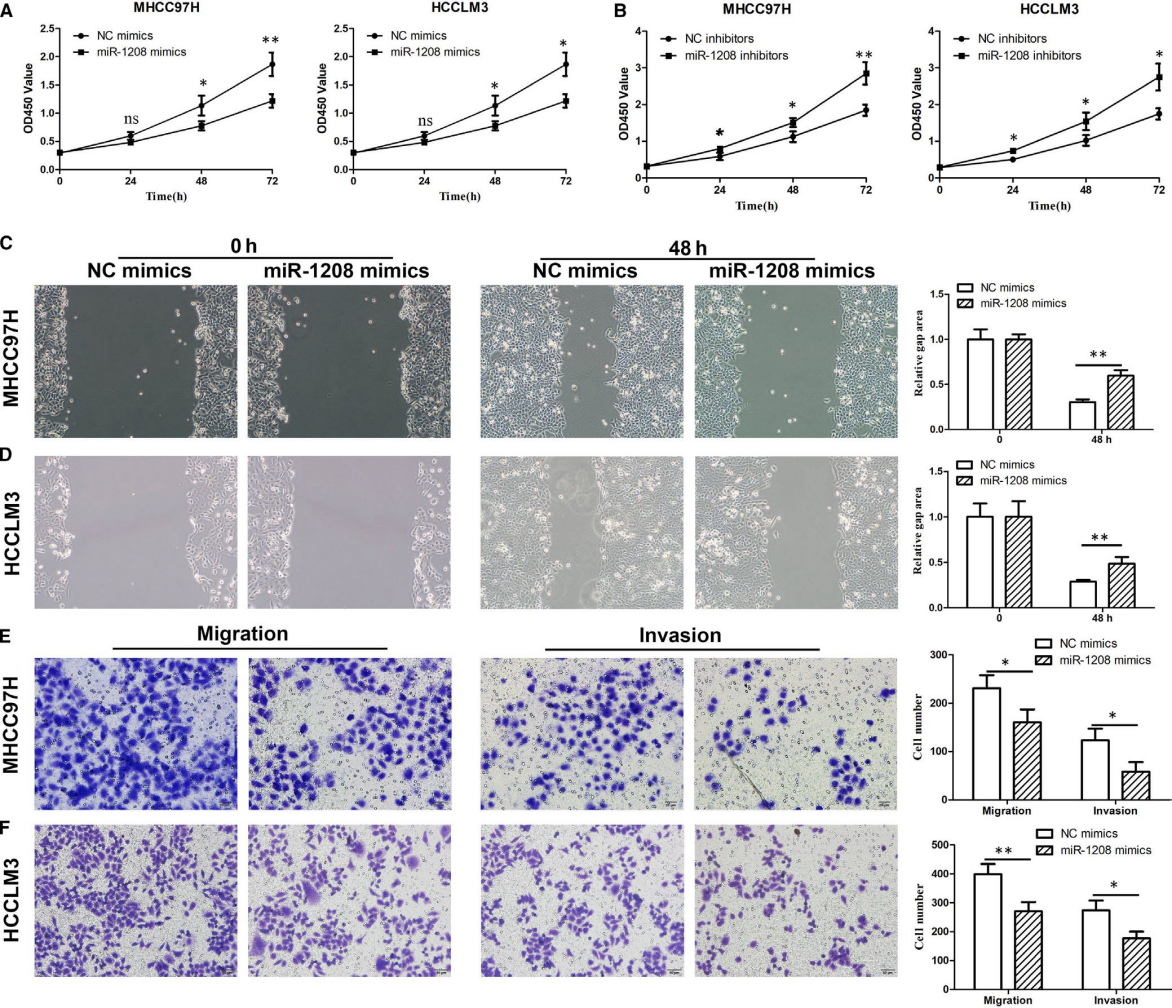
miR‐1208 inhibits HCC cells proliferation, migration and invasion. A and B, The proliferation of MHCC97H and HCCLM3 cells with repressing or overexpressing miR‐1208 was detected by CCK‐8 assay. C and D, Starch assay was performed to detect the migration of MHCC97H and HCCLM3 cells with repressing miR‐1208. E‐F, Cell migration and invasion were determined by Transwell assay after repressing miR‐1208. *P < .05, **P < .01 vs NC mimics or NC inhibitors. Ns, no significant

Increasing evidence showed that circRNA could function as a molecular sponge for miRNA. The target of circPUM1 was predicted by previous studies and Circular RNA Interactome website (https://circinteractome.nia.nih.gov/index.html). To confirm the binding miRNA of circPUM1 in HCC cells, we designed circPUM1‐specific probe (Bio‐circPUM1). RNA pull‐down assay showed that miR‐1208 was the most enriched miRNA in the captured fraction of c Bio‐circPUM1 compared with Bio‐NC in both MHCC97H and HCCLM3 cells (Figure 3A,B). We speculated that miR‐1208 might be the targets of circPUM1. MiR‐1208 has the assumed binding sites of circPUM1 (Figure 3C). To determine whether the predicted miR‐1208 binding site in circPUM1 sequence was essential for their interaction, the wild‐type (WT) and mutated (Mut) sequences of circPUM1 (in the predicted binding site) (Figure 3D) were inserted into luciferase reporter vector pmirGLO. The luciferase reporter assay showed that transfection of miR‐1208 mimics significantly repressed the luciferase activity of circPUM1, but it had no apparent effect on circPUM1 3′UTR mutant, suggesting that circPUM1 is directly targeted by miR‐1208 (Figure 3D). Anti‐Ago2 immunoprecipitation was performed in MHCC97H and HCCLM3 cells to study the relationship between miR‐1208 and circPUM1. We found that miR‐1208 mimics pulled down circPUM1 through anti‐AGO2 antibody not IgG (Figure 3E). Compared with mimic‐NC, the pulled down circPUM1 was enriched in HCC cells with miR‐1208 overexpression (Figure 3E). To further confirm the regulatory effect of circPUM1 on miR‐1208, sh‐circPUM1 or OE‐circPUM1 was transfected into HCC cells, respectively. QRT‐PCR arrays showed that circPUM1 could repress the expression of miR‐1208 in HCC cells (Figure 3F,G). Furthermore, we found that miR‐1208 could repress the expression of circPUM1 in HCC cells (Figure 4H). To investigate the expression of miR‐1208 in HCC, we performed qRT‐PCR in HCC cell lines and normal human liver cell line (THLE‐3). The result showed that circPUM1 was lowly expressed in MHCC97H and HCCLM3, compared with THLE‐3 and other HCC cell lines (Figure 3I). These results indicated that circPUM1 functions as a miR‐1208 sponge.

### 3.4 miR‐1208 suppresses migration and invasion of HCC cells

To investigate the function of miR‐1208 in HCC cells, we designed miR‐1208 mimics and inhibitors to overexpress or repress miR‐1208 (Figure S2). CCK‐8 assay showed that miR‐1208 mimics could inhibit HCC cell proliferation (Figure 4A). To further study the function of miR‐1208, we down‐regulated miR‐1208 by transfecting the miR‐1208 inhibitors into HCC cells. The results showed miR‐1208 inhibitors could promote HCC cell proliferation (Figure 4B). To detect the function of miR‐1208 in HCC cell migration, we performed scratch assays. The results showed that miR‐1208 mimics could inhibit MHCC97H and HCCLM3 cell migration (Figure 4C). In addition, we found that miR‐1208 mimics inhibit HCC cell migration and invasion by performing Transwell assays (Figure 4D). These results showed miR‐1208 played an anticancer role in HCC.

### 3.5 miR‐1208 mediates circPUM1‐dependent MAP3K2 expression in HCC cells

**FIGURE 5:**
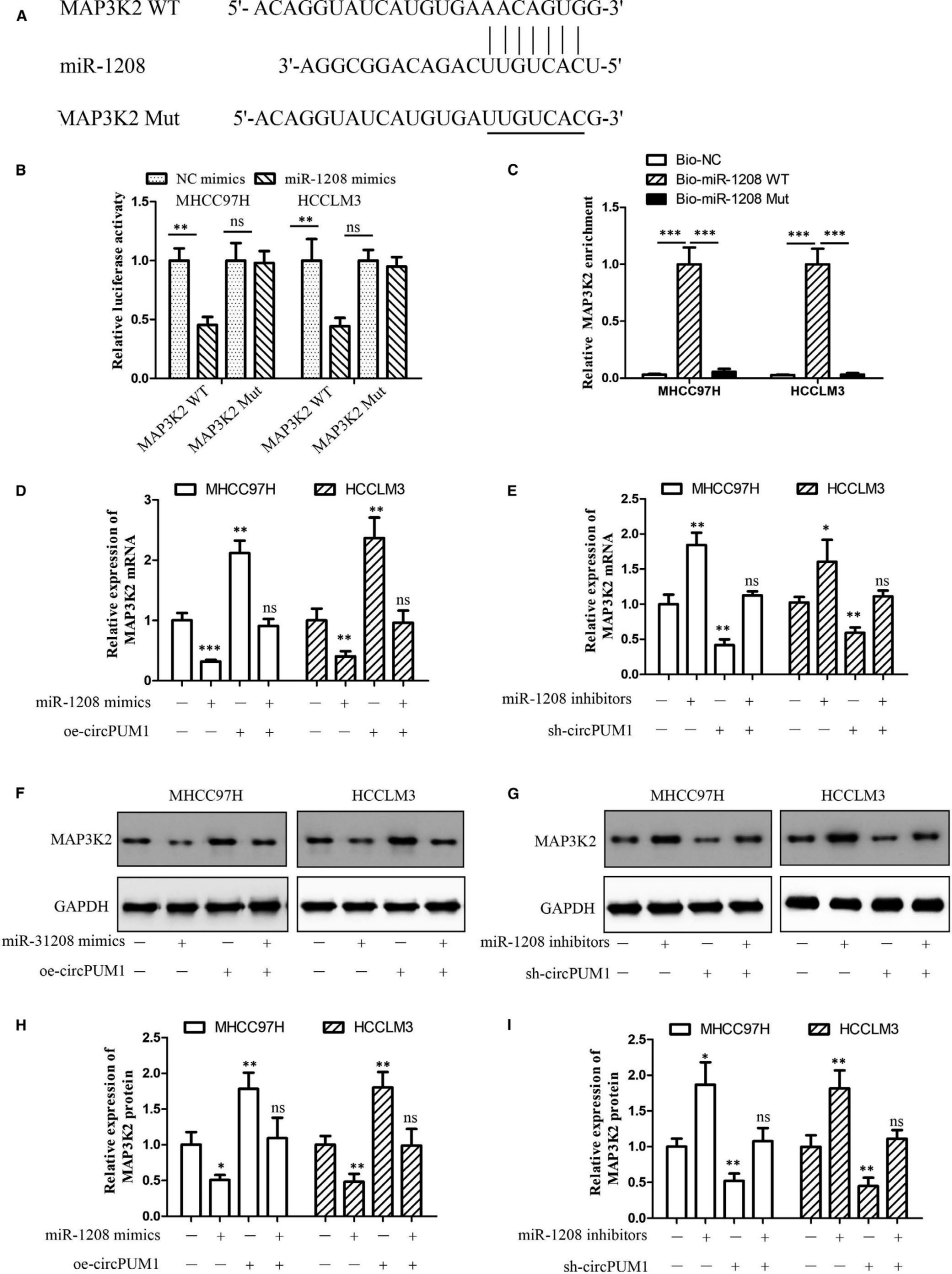
miR‐1208 represses MAP3K2 expression in HCC cells. A, Predicted binding sites of miR‐1208 in the 3’‐UTR of MAP3K2. B, Relative luciferase activity in MHCC97H and HCCLM3 cells after transfection of miR‐1208 mimics/NC or the 3’‐UTR of MAP3K2 WT/Mut. C, Relative expression of miR‐1208 binding MAP3K2 mRNA was examined by qRT‐PCR analysis by Bio‐miR‐1208 WT/Mut. D, Expression of MAP3K2 mRNA after miR‐1208 mimics or oe‐circPUM1 was transferred into MHCC97H and HCCLM3 cells by qRT‐PCR. E, Expression of MAP3K2 mRNA after miR‐1208 inhibitors or sh‐circPUM1 was transferred into MHCC97H and HCCLM3 cells by qRT‐PCR. F and H, Expression of MAP3K2 protein after miR‐1208 mimics or oe‐circPUM1 was overexpressed in MHCC97H and HCCLM3 cells. G and I, Expression of MAP3K2 protein after miR‐1208 or circPUM1 were repressed in MHCC97H and HCCLM3 cells. *P < .05, **P < .01, ***P < .001 vs NC. Ns, no significant

Bioinformatics analysis (http://www.targetscan.org/vert_72/) was used to predict that mitogen‐activated protein kinase kinase kinase 2 (MAP3K2) mRNA was the potential target of miR‐1208 (Figure S3). To confirm that miR‐1208 binds directly to MAP3K2 mRNA, we performed dual‐luciferase reporter assays. We constructed a wild‐type MAP3K2‐related luciferase reporter vector (MAP3K2‐WT) and a mutant MAP3K2‐related luciferase reporter vector (MAP3K2‐Mut), and tested luminescence under the influence of NC‐mimic, miR‐1208‐mimic and OE‐circPUM1 (Figure 5A). The luciferase reporter assay showed that transfection of miR‐1208 mimics significantly represses the luciferase activity of MAP3K2, which could be rescued by OE‐circPUM1. But they had no apparent effect on MAP3K2‐Mut, suggesting that MAP3K2 is directly targeted by miR‐1208 (Figure 5B). To test whether miR‐1208 could bind to MAP3K2 mRNA physically, we performed RNA pull‐down assay. MiR‐1208 probe, which is specifically antisense to spliced junction sequence and conjugated with biotin, was designed to enrich mRNAs which it targets (Figure 5C). QRT‐PCR arrays determined that MAP3K2 mRNA could interact with miR‐1208 via RNA pull‐down assay in HCC cells (Figure 5C). To further confirm the relationship between circPUM1, miR‐1208 and MAP3K2, we performed rescue experiments. Western blot analysis showed that miR‐1208 mimics can repress MAP3K2 mRNA and protein expression, which was up‐regulated by circPUM1 overexpression in HCC cells (Figure 5D, F and H). In addition, miR‐1208 inhibitors enhanced MAP3K2 mRNA and protein expression, which was repressed by circPUM1 shRNA in HCC cells (Figure 5E, G and I). Furthermore, we found that circPUM1 can regulate the protein expression of downstream target genes of MAP3K2 (Figure S4). These results demonstrated that circPUM1 could enhance MAP3K2 expression via sponging miR‐1208 in HCC cells.

### 3.6 circPUM1 promotes HCC cells development via altering miR‐1208/ MAP3K2 signals

**FIGURE 6:**
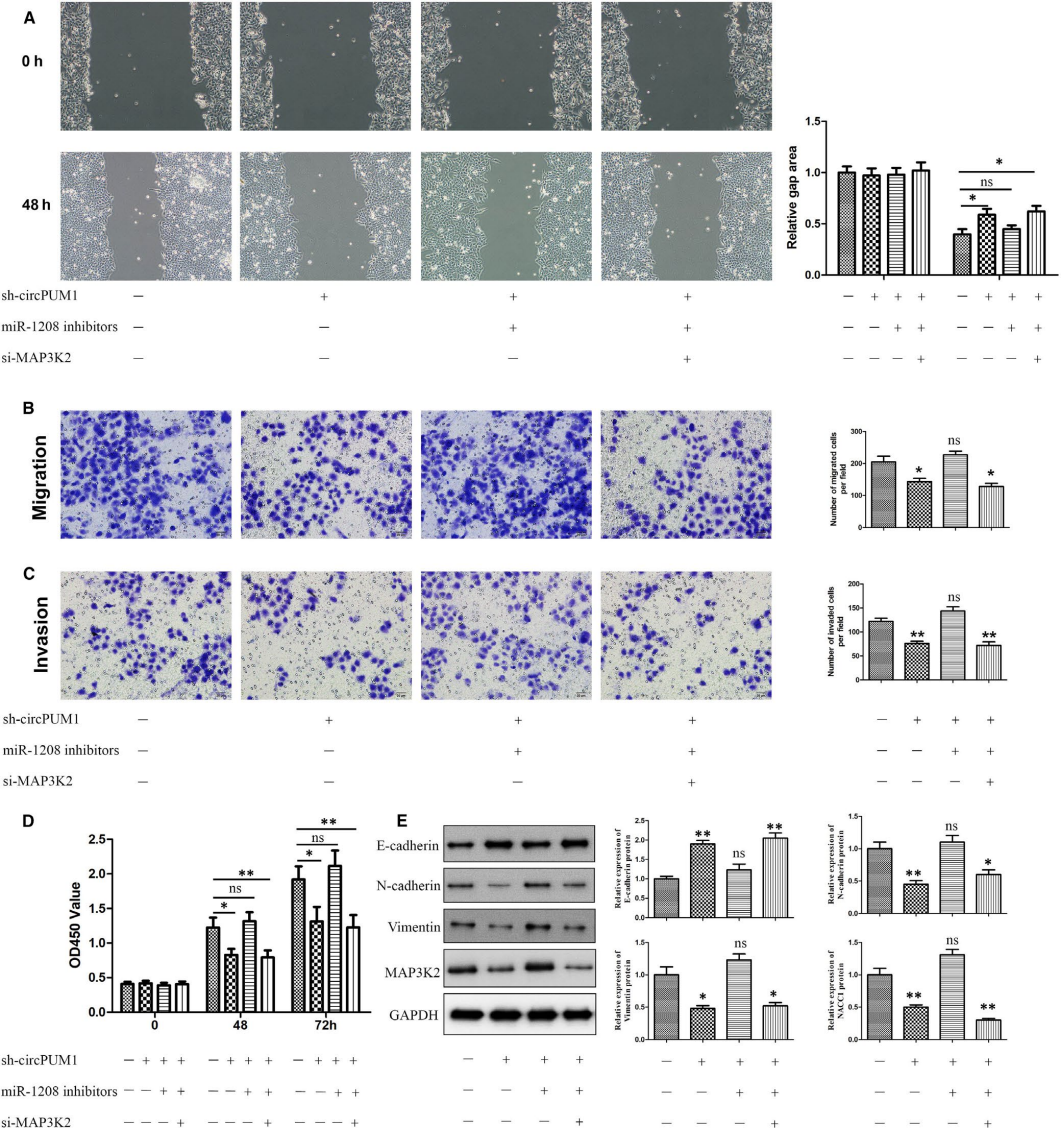
circPUM1 promotes HCC cells development via regulating miR‐1208/MAP3K2 axis. A, The migration of MHCC97H and HCCLM3 cells was detected by starch assay after miR‐1208, MAP3K2 or circPUM1 was repressed. B‐C, Transwell assay was performed to detect the migration and invasion of MHCC97H and HCCLM3 cells. D, CCK‐8 assay was performed to detect the proliferation of MHCC97H and HCCLM3 cells. E, Expression of E‐cadherin, N‐cadherin, Vimentin and MAP3K2 protein in MHCC97H and HCCLM3 cells was detected by Western blot. *P < .05, **P < .01 vs NC. Ns, no significant

In order to further study whether circPUM1 can promote the development of HCC by regulating miR‐1208/ MAP3K2 signals, we designed scratch, Transwell and CCK‐8 assays. Scratch assays showed that miR‐1208 inhibitors could eliminate the inhibition of sh‐circPUM1 on the migration of HCC cells, whereas MAP3K2 knockdown (MAP3K2 siRNA) could reverse this process (Figure 6A). In addition, similar trends were observed in Transwell (Figure 6B,C) and CCK‐8 assays (Figure 6D) as expected. EMT is closely related to tumour invasion, migration and metastasis, which is the main factor of poor prognosis of HCC.^17^ Western blot analysis showed that miR‐1208 inhibitors could eliminate the inhibition of sh‐circPUM1 on the N‐cadherin and Vimentin expression in HCC cells, whereas MAP3K2 knockdown reversed this process (Figure 6E). Furthermore, MAP3K2 siRNA could regulate the effect of sh‐circPUM1 and miR‐1208 inhibitors on E‐cadherin expression (Figure 6E). These results showed that circPUM1 may promote HCC cells migration, invasion and EMT by regulating miR‐1208/ MAP3K2 signals.

### 3.7 Inhibition of circPUM1 represses HCC tumour development in vivo

**FIGURE 7:**
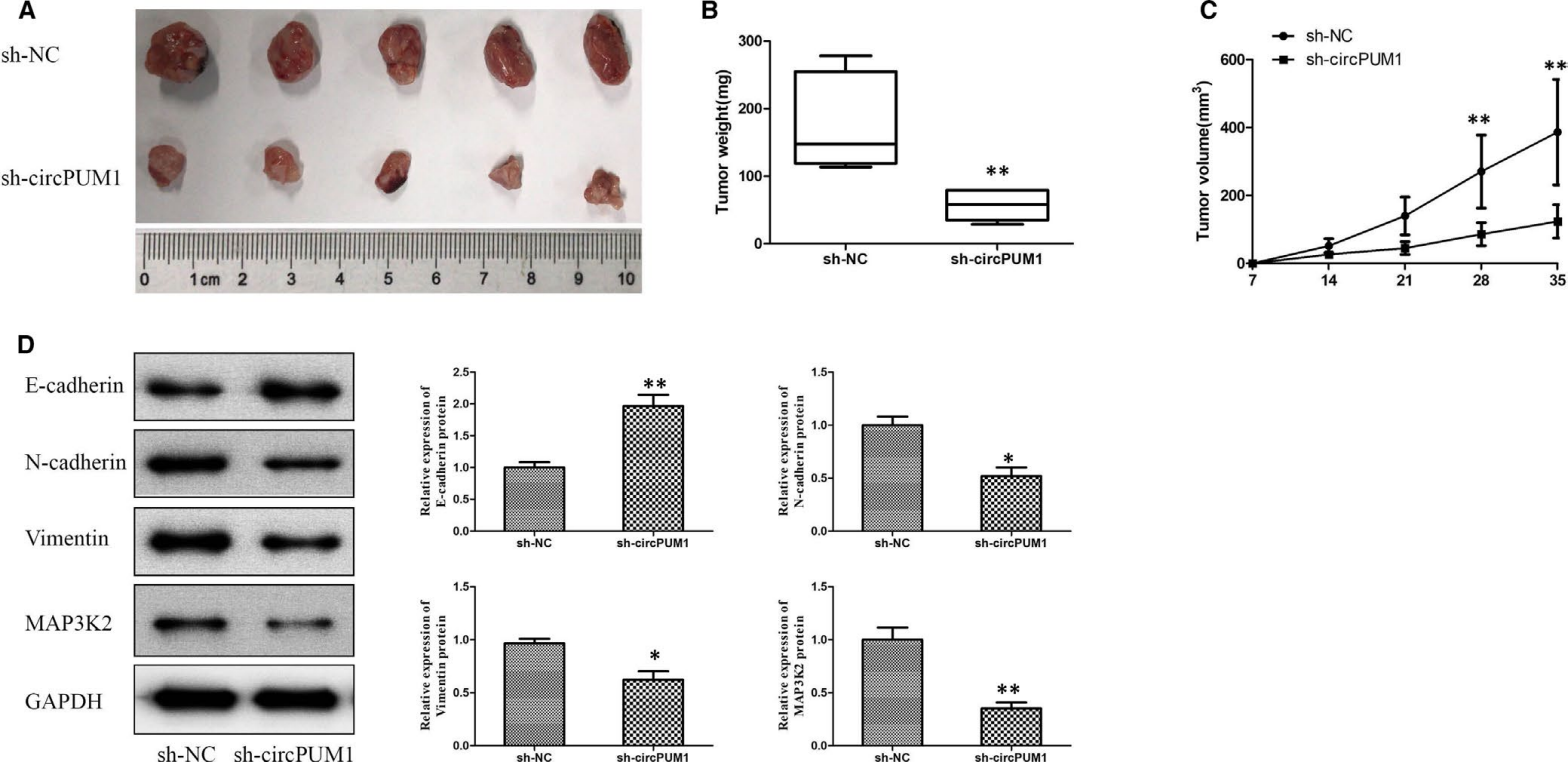
circPUM1 promotes HCC tumours development in vivo. MHCC97H cells stably silencing circPUM1 were inoculated subcutaneously into BALB/c nude mice. A, Images of xenograft tumours in nude mice. B, Tumour weight was measured in different groups. C, Tumour sizes were measured at different times. D, Expression of E‐cadherin, N‐cadherin, Vimentin and MAP3K2 protein in HCC tumours was detected by Western blot. *P < .05, **P < .01 vs NC

The effect of circPUM1 on HCC tumour development was verified with a tumour xenograft model which was generated by subcutaneous implantation of MHCC97H cells into nude mice. We found that circPUM1 knockdown inhibited tumour volume compared with the NC group (Figure 7A‐C). In addition, as expected, Western blot demonstrated circPUM1 knockdown could repress N‐cadherin, vimentin, MAP3K2 protein expression and up‐regulate E‐cadherin protein expression (Figure 7D), indicating that circPUM1 could promote the progress of EMT in HCC tumour. These results showed that inhibition of circPUM1 could repress the development of HCC tumour in vivo.

## 4 DISCUSSION

HCC is the main cause of cancer‐related death with high invasion and metastasis potential. The pathogenesis of HCC is complex, and its development involves many key signalling pathways.^18^ It is urgent to explore novel biomarkers for early diagnosis and prognosis of HCC, to enhance the survival rate and quality of life of HCC patients, as well as therapeutic strategies.^18^ MicroRNA (miRNA) is a category of small non‐coding RNA, which is associated with the proliferation, differentiation and development of tumour cells.^19^ CircRNA and mRNA could interact with microRNA to form a ceRNA network.^20^ Recent studies have found that circRNA regulate HCC progression of HCC as miRNA sponges and can be used as a target and biomarker for the treatment of HCC.^16^ In this study, we investigated the effect of circPUM1 on migration, invasion and migration of HCC in vitro and vivo. Our study suggested that circPUM1 promoted HCC progression by regulating miR‐1208/MAP3K2.

Currently, there are few reports on the function of circPUM1 in the development of mammalian diseases. Recent studies indicated that circPUM1 was highly expressed in endometrial cancer, lung adenocarcinoma, and ovarian cancer tissues.^11^, ^12^, ^14^ In addition, circPUM1 could inhibit tumour development via sponging microRNA.^11^, ^12^, ^14^ These studies suggest that circPUM1 may play an important role in the development of diseases such as tumours. In this study, we first report the role of circPUM1 in HCC. Our results showed that circPUM1 could promote the proliferation, invasion and migration of HCC in vitro. EMT is closely associated with tumour invasion and metastasis.^21^ CircPUM1 could promote the expression of EMT‐related proteins, such as Vimentin and N‐cadherin in vitro and in vivo. Our results indicated that circPUM1 might promote the invasion and metastasis of HCC tumour in vivo, but it requires further verification via animal experiments. Above all, our study suggested that circPUM1 could function as an oncogene in HCC.

Increasing evidence demonstrated that circRNAs can exert important biological functions by acting as miRNA sponges (inhibitors).^22^ Bioinformatics analysis indicated that miR‐1208 might be one of the targets of circPUM1. MiR‐1208 is encoded on chromosome 8q24. Previous studies showed that the expression of miR‐1208 is low expressed in breast cancer, Burkitt's lymphoma and colon cancer cell lines.^23^ However, in recent study, miR‐1208 was found to be up‐regulated in about 50% of gastric cancer tumours compared with adjacent non‐tumour tissues.^24^ Furthermore, Kim et al^25^ reported that miR‐1208 could enhance cisplatin sensitivity by targeting TBCK in renal cancer cells, which indicates that miR‐1208 may be a tumour suppressor gene. Therefore, the function of miR‐1208 in tumour is controversial, and the mechanism is rarely studied. It is necessary to further study the role and mechanism of miR‐1208 in tumours. Here, we reported the function of miR‐1208 in HCC for the first time. We demonstrated that miR‐1208 can inhibit the proliferation, invasion and metastasis of HCC by using miR‐1208 inhibitors and mimics in vitro. What's more, rescue experiments proved that circPUM1 regulates the progression of HCC through miR‐1208.

MicroRNA can partially or completely bind to the 3'UTR region of the target gene and then down‐regulate the expression of the target gene by reducing the stability or inhibiting translation of the target mRNA.^26^, ^27^ Bioinformatics analysis indicated that MAP3K2 might be the direct target of miR‐1208. MAP3K2, also called MEKK2, can regulate various tumour development‐related pathways, including β‐catenin, wnt, hedgehog.^28^, ^29^ Previous studies demonstrated that MAP3K2 activation could promote various tumour cells invasion and metastasis.^30^, ^31^ Therefore, MAP3K2 is a potential therapeutic target in tumour. In recent years, many miRNAs have been found to play an anticancer role by regulating MAP3K2. Zhang et al^32^ reported that MAP3K2, as a target of micRNA‐520b, can regulate cell proliferation in HCC cells. Jiang et al^33^ found that miR17/20a restoration could enhance the antitumour activity of NK cells via regulating MAP3K2 in solid tumours. In addition, circular RNA circ‐PITX1 was found to promote glioblastoma development by up‐regulating MAP3K2 via sponging miR‐379‐5p.^34^ Furthermore, miR‐302a could repress HCC cell proliferation via inhibiting MAP3K2 and FBX3.^35^ On the whole, however, the function of MAP3K2 in HCC progression is unclear. Through rescue experiments, we determined that circPUM1 promotes HCC migration, invasion and EMT is unclear via up‐regulating MAP3K2 which was inhibited by miR‐1208.

In conclusion, we studied the role and mechanism of circPUM1 in the development of HCC. We determined that circPUM1/miR‐1208/ MAP3K2 axis is an oncogenic pathway in HCC by promoting cell proliferation, invasion and migration.

## CONFLICT OF INTEREST

The authors declare that they have no competing interests.

## AUTHOR CONTRIBUTION

**Yaqiong Zhang:** Conceptualization (equal); Data curation (equal); Investigation (equal); Resources (equal); Writing‐original draft (equal); Writing‐review & editing (equal). **Dongguo Wang:** Methodology (equal); Writing‐original draft (equal). **Tao Zhu:** Formal analysis (equal). **Yu Jin:** Formal analysis (equal). **Xiaoyu Wu:** Data curation (equal); Investigation (equal); Writing‐original draft (equal). **Weidong Lin:** Software (equal); Validation (equal). **Mingqi Zhu:** Formal analysis (equal); Investigation (equal); Methodology (equal); Writing‐original draft (equal). **Yingjie Dai:** Project administration (equal); Writing‐original draft (equal). **Jie zhu:** Writing‐original draft (equal); Writing‐review & editing (equal).

## Supporting information

Figure S1

Figure S2

Figure S3

Figure S4

## Data Availability

The data used to support the findings of this study are available from the corresponding author upon request.
