# Supplementary figures and images for: CircPUM1 promotes hepatocellular carcinoma progression through the miR‐1208/MAP3K2 axis
Source: J Cell Mol Med. 2020 Dec 15;25(1):600–12. doi: 10.1111/jcmm.15998 (PMC7810943; doi:10.1111/jcmm.15998)

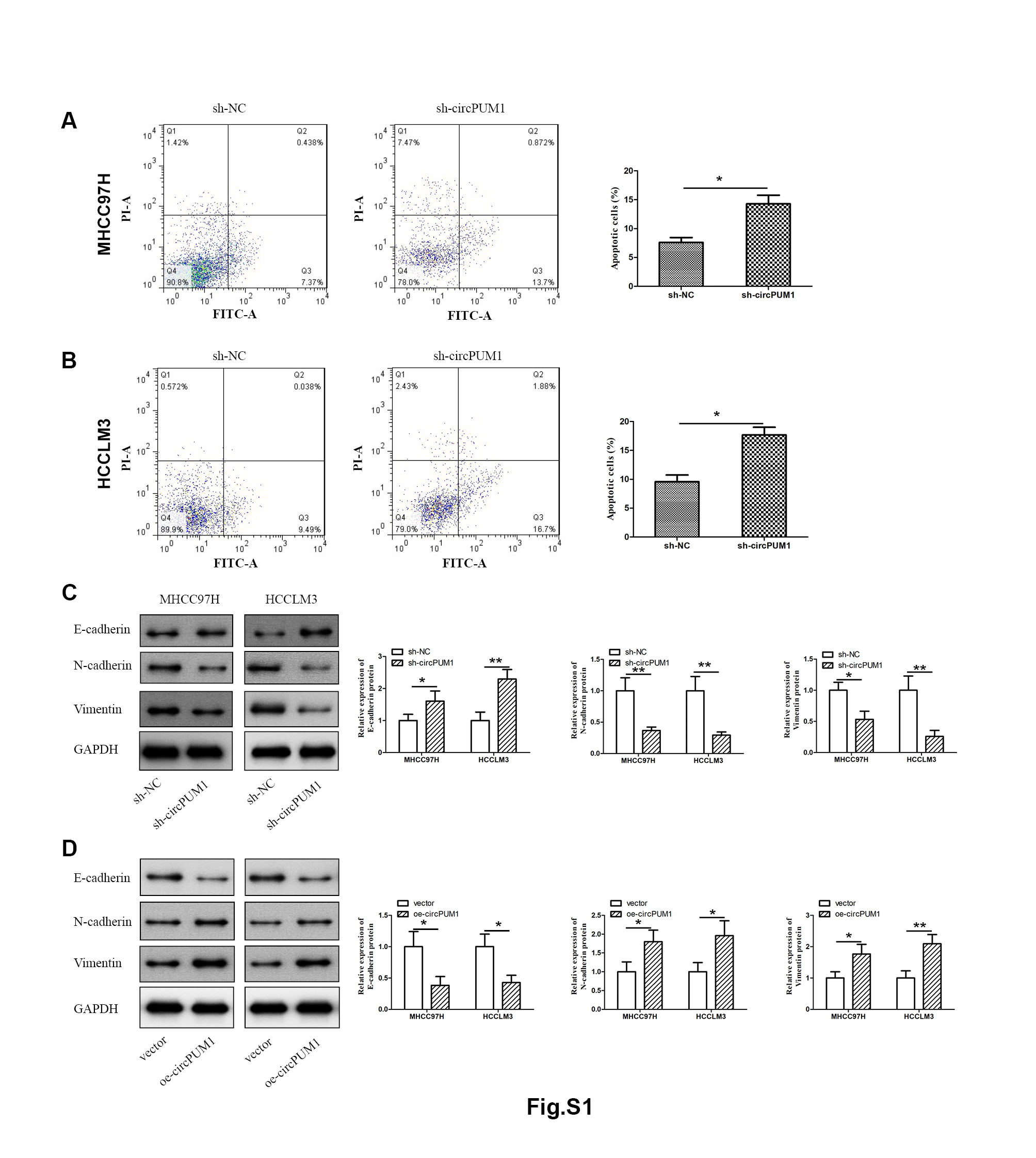

Supplement: Supplementary file 1 — Figure S1 [file JCMM-25-600-s001.jpg]

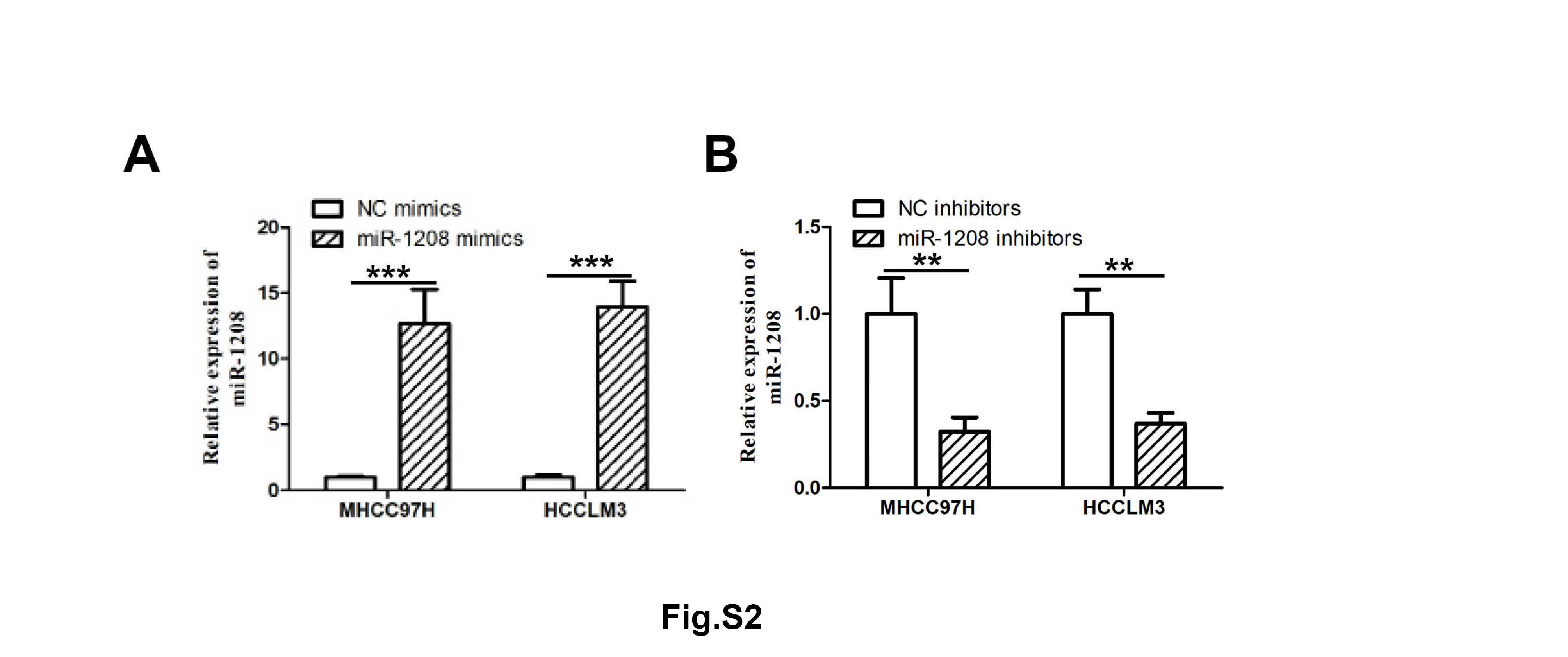

Supplement: Supplementary file 2 — Figure S2 [file JCMM-25-600-s002.jpg]

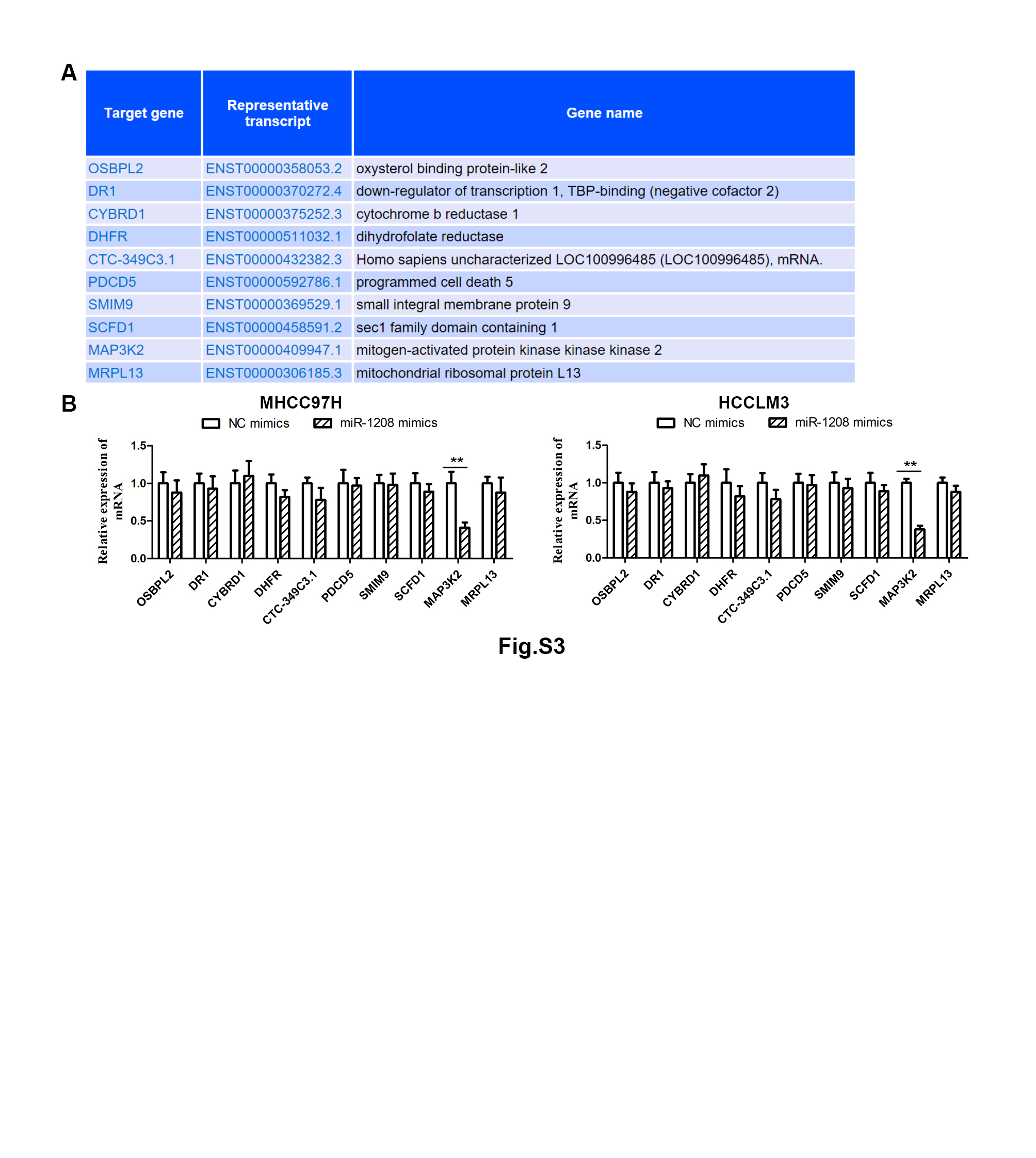

Supplement: Supplementary file 3 — Figure S3 [file JCMM-25-600-s003.jpg]

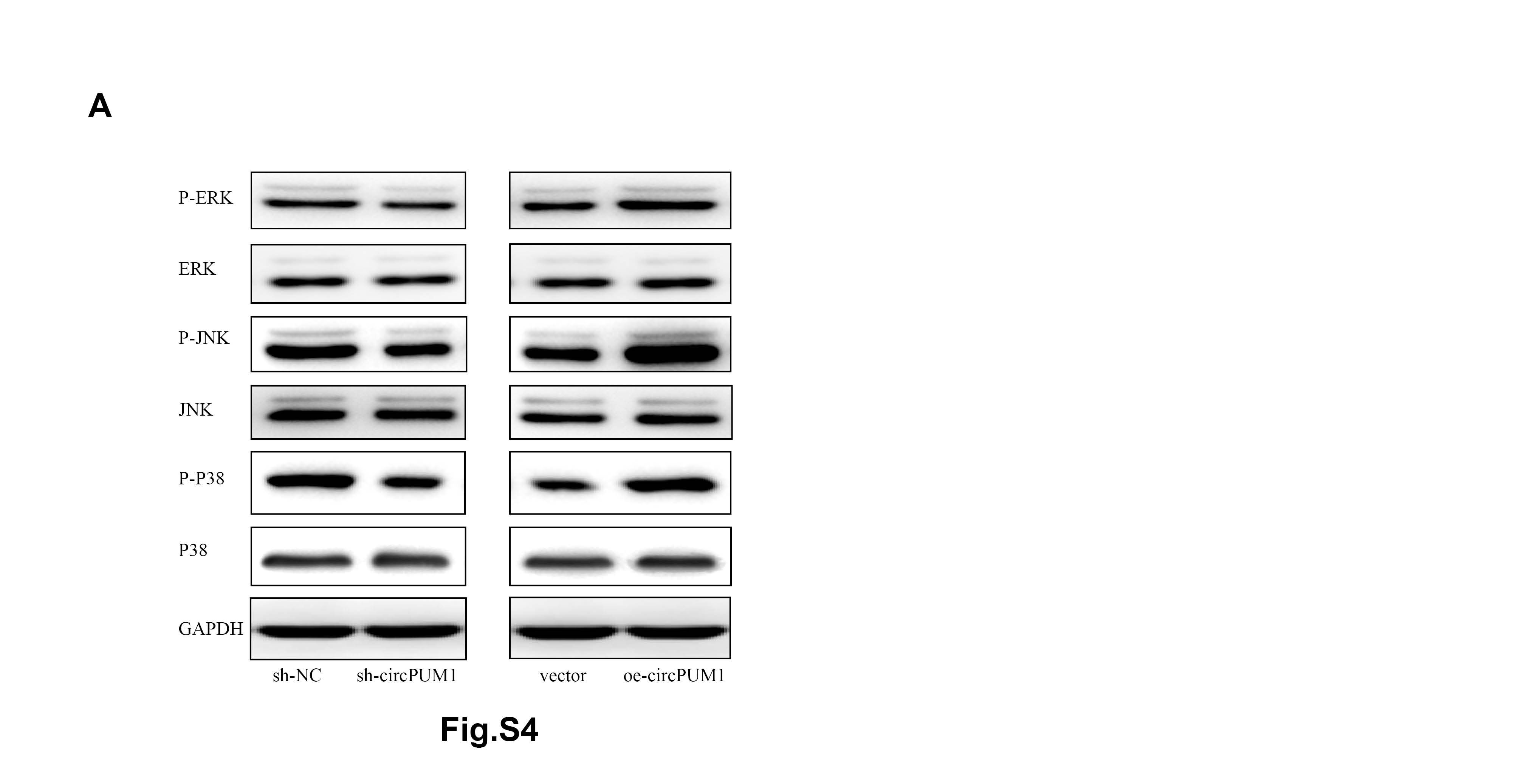

Supplement: Supplementary file 4 — Figure S4 [file JCMM-25-600-s004.jpg]
